# Supplementary material for: OpenMM 7: Rapid development of high performance algorithms for molecular dynamics
Source: PLoS Comput Biol. 2017 Jul 26;13(7):e1005659. doi: 10.1371/journal.pcbi.1005659 (PMC5549999; doi:10.1371/journal.pcbi.1005659)
Supplement: S1 Source Code — (ZIP) [file pcbi.1005659.s002.zip › OpenMM-7.0.1-Source/docs/api-python/generated/simtk.openmm.openmm.Discrete1DFunction.html]

Discrete1DFunction — OpenMM 7.0.1.dev-5e86c4f documentation


# Discrete1DFunction¶

*class* `simtk.openmm.openmm.``Discrete1DFunction`(*values*)¶
:   This is a TabulatedFunction that computes a discrete one dimensional function f(x). To evaluate it, x is rounded to the nearest integer and the table element with that index is returned. If the index is outside the range [0, size), the result is undefined.

    `__init__`(*self*, *values*) → Discrete1DFunction¶
    :   This is a TabulatedFunction that computes a discrete one dimensional function f(x). To evaluate it, x is rounded to the nearest integer and the table element with that index is returned. If the index is outside the range [0, size), the result is undefined.

    Methods

    |  |  |
    | --- | --- |
    | `Copy`((self) -> Discrete1DFunction) |  |
    | `__init__`((self, values) -> Discrete1DFunction) | This is a TabulatedFunction that computes a discrete one dimensional function f(x). |
    | `getFunctionParameters`(self) |  |
    | `setFunctionParameters`(self, values) |  |

    `getFunctionParameters`(*self*)¶

    `setFunctionParameters`(*self*, *values*)¶

    `Copy`(*self*) → Discrete1DFunction¶

    `__delattr__`¶
    :   x.\_\_delattr\_\_(‘name’) <==> del x.name

    `__format__`()¶
    :   default object formatter

    `__getattribute__`¶
    :   x.\_\_getattribute\_\_(‘name’) <==> x.name

    `__hash__`¶

    `__reduce__`()¶
    :   helper for pickle

    `__reduce_ex__`()¶
    :   helper for pickle

    `__sizeof__`() → int¶
    :   size of object in memory, in bytes

    `__str__`¶

High performance molecular simulation on GPUs


### Navigation

- Application Layer
- Library Layer
  - Core Objects
  - Forces
  - Integrators
  - Extras

©2015, Stanford University and the Authors.
|
Powered by Sphinx 1.3.1
& Alabaster 0.7.7
|
Page source
